# Evaluation of lactulose synthesis using immobilized β-galactosidase in different reactor configurations

**DOI:** 10.1007/s00449-026-03349-z

**Published:** 2026-05-23

**Authors:** Carla Cristina de Sousa, Amanda Carmelo da Rocha, Eloízio Júlio Ribeiro, Larissa Nayhara Soares, Santana Falleiros, Miriam Maria de Resende

**Affiliations:** Chemical Engineering Faculty, Federal University of Uberlândia, P.O. Box 593, Av. João Naves de Ávila 2121, Campus Santa Mônica, Bloco 1K, Uberlândia, MG 38408- 144 Brazil

**Keywords:** Transgalactosylation, Prebiotics, Immobilization, Operational conditions

## Abstract

Lactulose is a disaccharide composed of galactose and fructose. In the human body, it functions as a prebiotic, promoting the growth of beneficial bacteria, such as bifidobacteria and lactobacilli, in the gastrointestinal tract. Lactulose is produced via a transgalactosylation reaction between lactose and fructose, catalyzed by β-galactosidase. Prominent β-galactosidases used in lactulose synthesis include those from *Aspergillus oryzae* and *Kluyveromyces lactis*. Various methodologies have been developed to enhance the efficiency of enzymatic lactulose production. The objective of this study was to produce lactulose using β-galactosidase from *K. lactis* and *A. oryzae*, in both free and immobilized forms, and to evaluate operational conditions in different reactor configurations. Sugar concentrations in the medium and the lactose-to-fructose ratio were assessed to optimize lactulose synthesis. Experiments were conducted sequentially in a benchtop conical reactor with orbital stirring, a stirred-tank reactor with magnetic stirring, and a fixed-bed reactor. Sugar concentrations were determined using high-performance liquid chromatography (HPLC). Of the enzymes tested, only silica-immobilized *K. lactis* β-galactosidase failed to produce a substantial lactulose yield. All three reactor configurations proved suitable for lactulose production. Under the evaluated conditions, lactulose concentrations ranged from 19 to 30 g·L⁻¹. Among the tested systems, the stirred-tank reactor with magnetic stirring showed the most promising performance for lactulose production. These results highlight the potential of the enzymatic process for lactulose synthesis and provide valuable insights for future studies on the production of this prebiotic.

## Introduction

Lactulose, a non-digestible disaccharide, has various applications in the food and pharmaceutical industries. Because of its ability to promote the growth of bifidobacteria, it has become a popular ingredient in functional foods designed to regulate the intestines and support overall health [1, 2]. It can be produced through the isomerization of lactose, which can be chemically catalyzed [3–5] or via an enzymatic process involving the transgalactosylation of fructose and lactose [6, 7]. Due to the operational conditions, the enzymatic route has emerged as a promising alternative for producing this disaccharide. In this context, the use of enzymes such as β-galactosidase has been investigated to develop production processes that align with the principles of green chemistry. These principles include high specificity, selectivity and catalytic activity under mild environmental conditions [1, 8, 9].

The enzyme β-galactosidase is a hydrolase, which catalyzes the hydrolysis of lactose into β-D-galactose and α-D-glucose, as well as catalyzing the transgalactosylation reaction. In this reaction, lactose acts as both a donor and an acceptor of galactosyl units for di-, tri-, and other higher-formula GOS. β-galactosidase can be produced by different microorganisms, particularly yeasts and fungi. For applications in the food industry, enzymes must not pose risks to consumer health. The β-galactosidases produced by *K. lactis* and *A. oryzae* are classified as Generally Recognized as Safe (GRAS) by the Food and Drug Administration (FDA). In addition, these enzymes present other technological advantages, showing good performance in lactose hydrolysis and in the production of galactooligosaccharides. β-galactosidases produced by *K. lactis* exhibit high specificity for lactose and have a neutral pH optimum, close to the pH of milk. In contrast, those produced by *A. oryzae* have a more acidic optimal pH, typically between 4.5 and 5.0, are more resistant to higher temperatures, and are produced extracellularly, which facilitates their extraction [10, 11].

Using enzymes in their natural form can present difficulties, such as ensuring stability in terms of temperature and pH during the process. To overcome these problems, immobilization methodologies are being applied. There are various immobilization methods. One method is physical adsorption, which is a simple and inexpensive process that promotes soft binding of the enzyme to the support. Covalent bonding promotes a strong bond between the enzyme and the support matrix. This improves the resistance of immobilized enzymes to extreme physical and chemical conditions while reducing losses due to leaching [12].

In this context, using immobilized β-galactosidase improves lactulose synthesis by reducing the amount of required enzyme, making it a promising alternative to the current chemical process. The operational stability of this enzymatic process depends on the reaction medium, the biocatalyst, and the reactor configuration, particularly when scaling up to an industrial level. Many studies are being applied with the aim of improving lactulose synthesis in different processes and immobilization methods. Girão-Neto et al. [13] used *K. lactis* β-galactosidase, which was immobilized on various supports, to synthesize lactulose. Ramírez et al. [14] evaluated the enzymatic production of lactulose using *A. oryzae* β-galactosidase immobilized on glyoxyl agarose in fed-batch and repeated-fed-batch reactors. Serey et al. [15] studied lactulose production in batch and repeated-batch operations using *A. oryzae* β-galactosidase immobilized on agarose and quaternary ammonium.

New studies are seeking alternatives for lactulose synthesis, seeking more efficient processes. Thus, the objective of this study was to produce lactulose using β-galactosidase from *K. lactis* and *A. oryzae*, both in free and immobilized forms. The study evaluated the operational conditions in different reactor configurations to optimize the cost-benefit ratio of the enzyme production process.

## Materials and methods

### Materials

The enzymes used were β-galactosidase Lactozyme 2600 L from *K. lactis* with an activity of 2600 U/g and β-galactosidase from *A. oryzae* with an activity of 8.0 U/mg of solids, both of which were obtained from Sigma-Aldrich Chemical Co. We used controlled porosity silica, obtained from Corning Glass Works, with an average pore diameter of 375 Å, and Duolite^®^ A-568 ion exchange resin, supplied by Rohm and Haas Company, as immobilization supports. Standard reagents, including lactulose, glucose, lactose, galactose, and fructose, were obtained from Sigma-Aldrich Chemical Co. A commercial galactooligosaccharide with a 90% concentration was obtained from Nutramax – Life Science Products. All other reagents were of analytical grade.

### Methods

#### Activity of the free and immobilized enzyme

The enzymatic activity of the enzymes in their free and immobilized forms was determined using the initial rate method for the lactose hydrolysis reaction. The reaction occurred in a 75 mL mixed-batch reactor containing a 50 g.L^-1^ lactose (PA) solution prepared in a pH 7.0 lactic acid buffer at 30 °C [16]. In each experiment, 5 mL of the enzyme solution was added to the reactor to determine the activity of the free enzyme. Then, a stainless-steel basket containing either 0.1 g of enzyme immobilized on silica or 0.5 g of enzyme immobilized on Duolite^®^ A-568 ion-exchange resin was added to the reactor for the immobilized enzyme determination. One unit of activity (U) was defined as the amount of glucose produced per minute (µmol/min). The glucose oxidase method was used to determine the glucose formed [17].

#### Determination of sugar concentration

Sugar concentrations were determined using high-performance liquid chromatography (HPLC) on a Shimadzu LC-20 A Prominence system with a Supelco C18 column. The samples were diluted, filtered, and injected. The carrier solution was deionized water with a flow rate of 0.5 mL.min^-1^, an oven temperature of 80 °C, and an injection volume of 20 µL. Sugar concentrations were calculated using previously prepared standard curves. The standards used were lactulose, lactose, glucose, galactose, fructose, raffinose, stachyose (all from Sigma Aldrich Brasil), and a 90% concentrated commercial galacto-oligosaccharide, kindly provided by Nutramax – Life Science Products.

### Immobilization of β-galactosidase

The β-galactosidase from *K. lactis* was immobilized on controlled-porosity silica that was previously functionalized with 0.6% 3-aminopropyl triethoxysilane (APTES) in an aqueous solution. After the functionalization of the silica, the samples were activated with a glutaraldehyde cross-linker at a concentration of 6.86%, prepared in a 0.2 M sodium phosphate buffer at pH 7.0. The activated silica was then used to immobilize the β-galactosidase using a 0.1 g solution of the enzyme, prepared at a concentration of 0.81% (v.v^− 1^) in lactic buffer at pH 7.0. The samples were agitated at 120 rpm in a rotary incubator at 23 ± 1 °C for 6 h [16].

According to the manufacturer’s methodology, *A. oryzae* β-galactosidase was immobilized on previously activated Duolite ^®^ A-568 resin using a 1:10 solution of 1 M hydrochloric acid and 1 M sodium hydroxide for 30 min in a rotary incubator at 50 rpm. The resin was then washed with distilled water, vacuum filtered and dried at room temperature. The enzyme was immobilized by adsorption on the activated resin with Duolite ^®^ A-568 ion-exchange resin. The resin had a mass of 0.5 g per 10 mL of a 5.0 g.L^− 1^ enzyme solution, which was prepared in sodium acetate buffer at pH 4.5. The samples were kept under agitation at 120 rpm in a rotary incubator at 25 ± 1 °C for two hours [18].

### Lactulose synthesis

The synthesis of lactulose was evaluated in different reactor configurations. The sugars produced were quantified in all production runs. The lactulose production yield (Y_Lu_) was defined as the ratio of the produced lactulose concentration to the initial lactose concentration (Eq. 1). Lactose conversion was also evaluated using Eq. 2.

1 $$\:{Y}_{Lu}=\:\frac{concentration\:of\:lactulose\:produced}{initial\:lactose\:concentration}$$

2 $$\:{C}_{Lac}=\:\frac{{LAC}_{initial}\:-\:{LAC}_{final}}{{LAC}_{initial}}$$

#### Synthesis of lactulose in a benchtop conical reactor with an orbital stirrer

The synthesis of lactulose was initially evaluated in a 125 mL benchtop conical reactor with orbital stirring using free and immobilized β-galactosidases from *K. lactis* and *A. oryzae*. The initial concentration of sugars in the medium was 500 g.L^− 1^, and it has a 1:2 ratio of lactose to fructose. The conditions were 40 °C and 120 rpm for six hours. These conditions were based on literature studies as a starting point [13, 19]. For the β-galactosidases from *K. lactis*, a 0.05 M potassium phosphate buffer at pH 7.0 was used. For β-galactosidases from *A. oryzae*, a 0.2 M acetate buffer at pH 4.5 was used.

The initial sugar concentration of 200 g.L^− 1^ was evaluated with the β-galactosidase that produced the best results. The synthesis was conducted under the same conditions: a 1:2 ratio of lactose to fructose, 40 °C, 120 rpm, and a six-hour reaction time.

Later, the ratio of lactose to fructose was evaluated in the production of lactulose using both free and immobilized β-galactosidase from *A. oryzae*. The reactions were performed using lactose and fructose dissolved in a 0.2 M sodium acetate buffer at pH 4.5. The initial total carbohydrate concentration was 500 g.L^− 1^, with the lactose-to-fructose ratio varying between 1:1 and 1:2. The reactions were conducted at 50 °C and 120 rpm for three hours. Following this, the produced sugars were quantified, and the lactulose yield (Eq. 1) and lactose conversion (Eq. 2) were calculated.

#### Synthesis of lactulose in a jacketed, stirred tank reactor with magnetic stirring

**Fig. 1:**
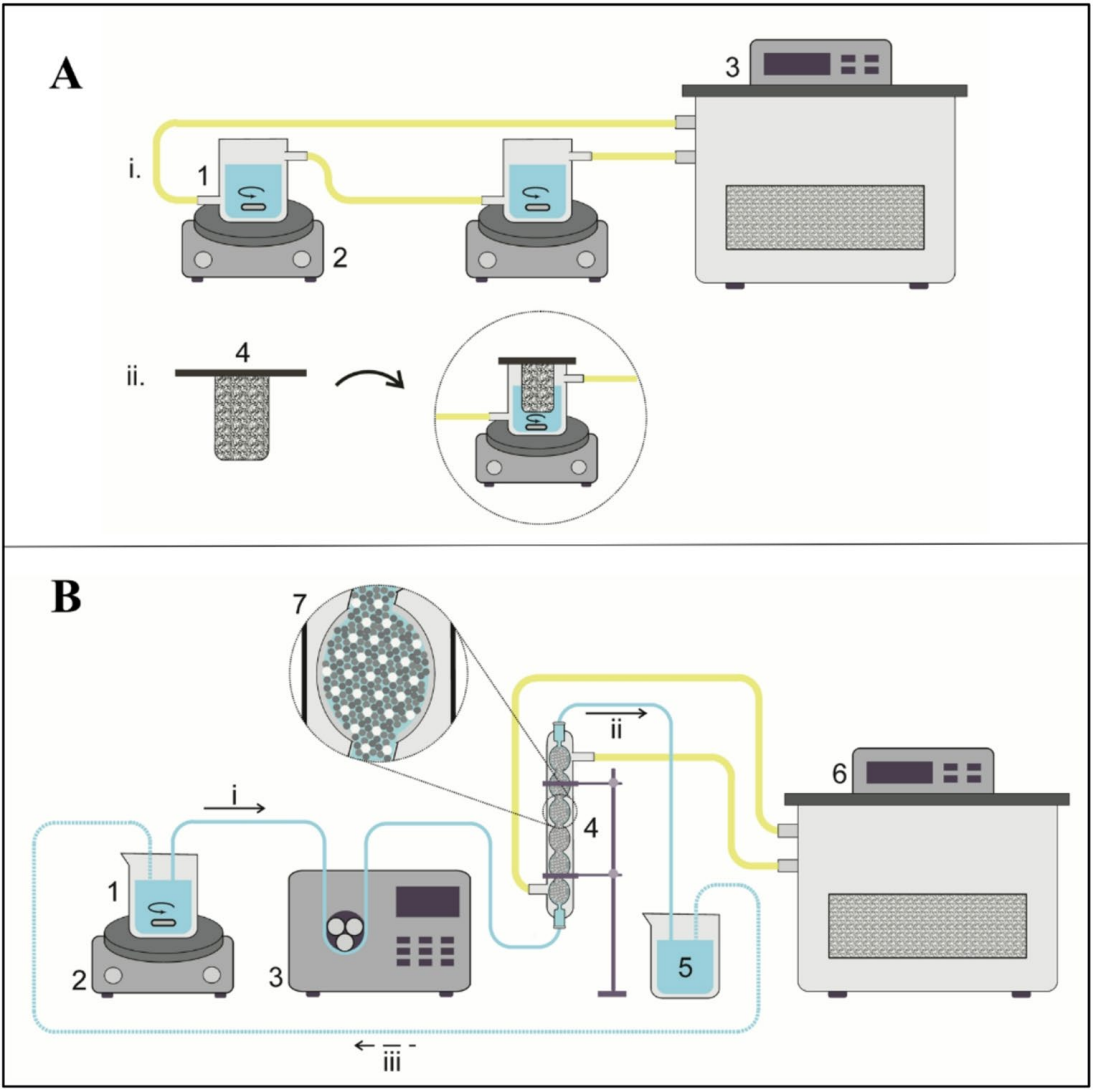
**A** Diagram showing the process of lactulose in a jacketed stirred tank reactor with a magnetic stirrer. 1- Jacketed reactor; 2- Magnetic stirrer; 3- Thermostatic bath; 4- Stainless steel basket. (i) Reaction with free enzyme; (ii) Reaction with immobilized enzyme. **B** Schematic of lactulose synthesis in a fixed bed reactor: 1- Feed solution; 2- Magnetic stirrer; 3- Peristaltic pump; 4- Column packed with immobilized biocatalyst; 5- Product; 6- Thermostatic bath; 7- Column packing (resin and glass beads). (i) Reactor feed line. (ii) Product production line. (iii) Product recycling line

Another reactor configuration evaluated was a jacketed stirred-tank reactor with magnetic agitation. In this configuration, the rotational speed of the reaction system could be increased. The reactions were carried out in an experimental unit composed of two jacketed reactors connected in series to a thermostatic bath (Fig. 1A). Each reactor had a working volume of 50 mL and was equipped with an external water-circulation jacket to maintain the temperature. Agitation was provided using magnetic stirrers. For reactions involving the immobilized enzyme, a stainless-steel basket was used, in which the immobilized biocatalyst was placed to minimize friction with the magnetic stir bar.

This study evaluated lactulose production using free and immobilized β-galactosidases from *K. lactis* and *A. oryzae*. Reactions were carried out in a medium containing 500 g·L⁻¹ of initial sugars with a lactose to fructose ratio of 1:2. Reaction temperatures were set at 40 °C and 50 °C for the β-galactosidases from *K. lactis* and *A. oryzae*, respectively, according to their optimal temperatures. The reaction time was 3 h, based on previous experiments. After the reaction period, the produced sugars were quantified, and lactulose yield (Eq. 1) and lactose conversion (Eq. 2) were calculated.

#### Synthesis of lactulose in a fixed-bed reactor

A fixed-bed reactor was used to evaluate the synthesis of lactulose. The column was equipped with a heating jacket and had a working volume of 42.98 mL and measured 16.20 cm in height and 1.84 cm in diameter. The reactor operated continuously in an upward-flowing state. A peristaltic pump was used to feed the heated lactose and fructose solution under constant magnetic stirring. Figure 1B shows the process schematic.

Given the size of the column, an inert material was used alongside the immobilized biocatalyst mass. Two-millimetre glass beads, which had been previously washed with distilled water, were adopted as the inert material. The inert material consisted of 5.0 g of enzyme immobilized on Duolite ^®^ A-568 resin and 30.0 g of glass beads.

*A. oryzae* β-galactosidase was immobilized in accordance with the procedure outlined in Sect.  2.3. The process was conducted at 50 °C using a 500 g.L^− 1^ sugar solution as the feed substrate with a lactose-to-fructose ratio of 1:2. The solution was prepared in a 0.2 M sodium acetate buffer at pH 4.5. The feed flow rate in the fixed-bed reactor was set at 1.0 mL.min^− 1^. The process was conducted continuously for one hour. During this time, samples were collected at defined intervals and transferred to a boiling bath for ten minutes to stop the enzymatic reaction. These samples were then diluted and filtered, after which carbohydrate quantification was performed.

**Fig. 2:**
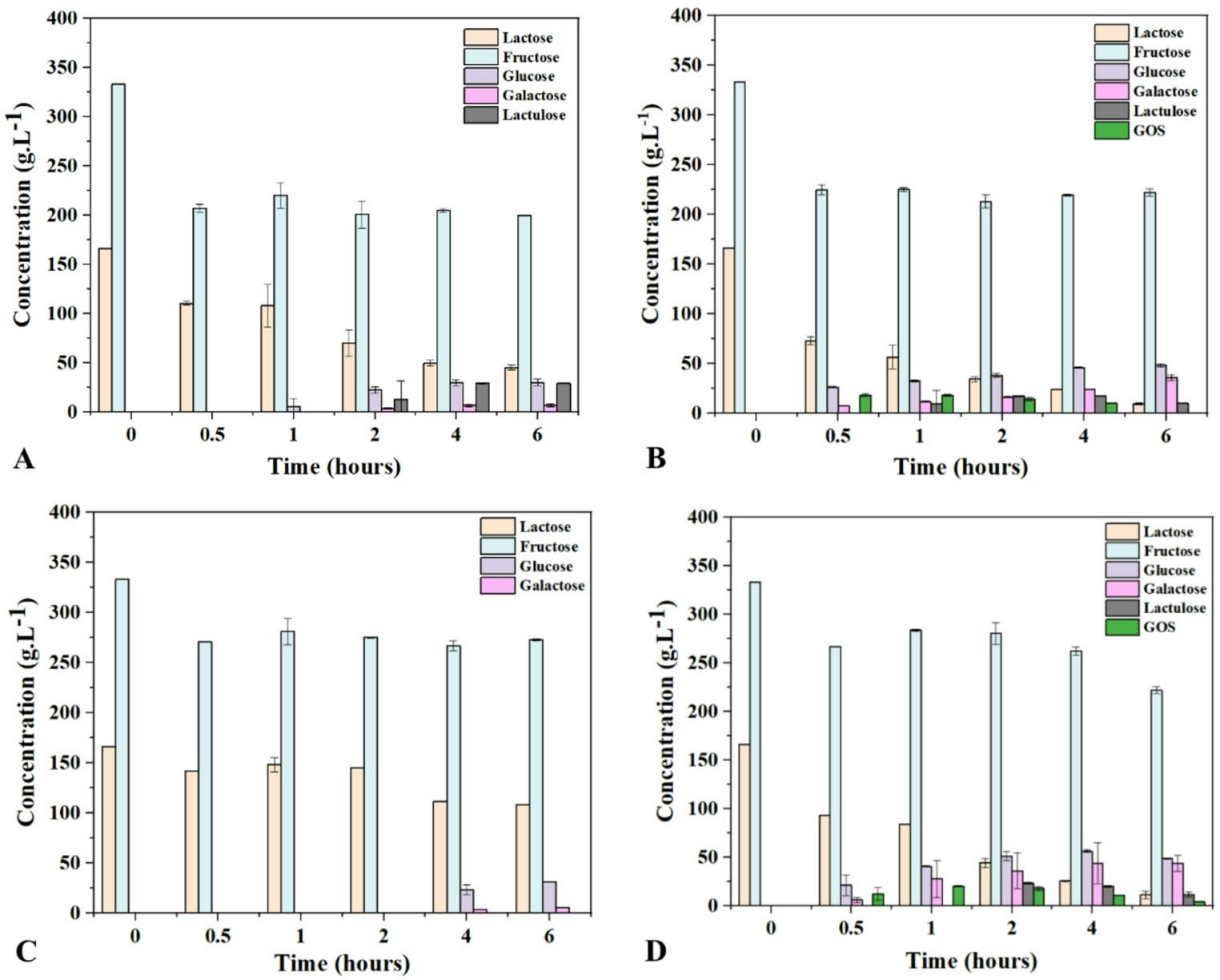
Concentration of products formed in the reaction at 40 °C and 120 rpm A free β-galactosidase from *K. lactis*; **B** free β-galactosidase from *A. oryzae*; **C** β-galactosidase from *K. lactis* immobilized in controlled porosity silica; **D** β-galactosidase from *A. oryzae* immobilized in Duolite ^®^ A-568 resin

At the end of the continuous process, a product recycling step was initiated (Fig. 2 – iii). In this step, the reactor outlet stream was directed to the feed reservoir. Consequently, the feed stream contained both the sugar solution and the reactor product. After 3 h of reaction under product recycling conditions, the samples were analyzed.

### Statistical analysis

All analyses were performed in duplicate. When necessary, the Tukey test for the least significant difference (*p* < 0.05) between the means was performed using the Sisvar 5.6 statistical program. The Origin Pro 2018 software was used to create the graphs.

## Results and discussion

### Synthesis of lactulose in a benchtop conical reactor with an orbital stirrer

Lactulose was synthesized using β-galactosidases from *K. lactis* and *A. oryzae*, both in their free and immobilized forms. The transgalactosylation reaction that produces lactulose occurs concurrently with lactose hydrolysis. Thus, other sugars, such as glucose and galactose, as well as different types of galacto-oligosaccharides (GOS), are also formed. Figure 2 shows the products of the reaction using free β-galactosidases.

Figure 2A and B illustrate the consumption of lactose and fructose during the reaction. Lactose undergoes greater reduction because it participates in the transgalactosylation process for synthesizing lactulose and can be hydrolyzed into glucose and galactose through concurrent hydrolysis.

Free *K. lactis* β-galactosidase product formation began after one hour and increased up to six hours of reaction. Lactulose, the desired product, was only synthesized after two hours, reaching its maximum production at six hours, with a concentration of 29.18 ± 0.55 g·L⁻¹. By the end of the reaction, 72.78% of lactose had been converted. The formation of glucose and galactose also increased throughout the reaction. Other GOS were not detected under these conditions.

For β-galactosidase from *A. oryzae* (Fig. 2B), product formation was observed after 0.5 h of reaction. Lactulose was therefore formed after one hour and reached its maximum concentration at two hours, with 17.20 ± 0.57 g·L⁻¹. After four hours, the lactulose concentration remained practically constant at 17.03 ± 0.24 g·L⁻¹, and by the end of the reaction, it had decreased to 10.14 ± 0.44 g·L⁻¹. The formation of glucose and galactose, the hydrolysis products, continued to increase until six hours of reaction. Lactose conversion reached 94.41%. GOS were also detected between 0.5 and 4 h of reaction.

When comparing lactulose production by β-galactosidase from *K. lactis* and *A. oryzae* in their free forms, the *K. lactis* enzyme exhibited a higher lactulose concentration (29.18 g·L⁻¹). However, this enzyme required a longer reaction time to reach its maximum yield. In contrast, the β-galactosidase from *A. oryzae* achieved its maximum lactulose concentration (17.20 g·L⁻¹) within only two hours of reaction. Both enzymes demonstrated promising potential for lactulose synthesis under the evaluated conditions. The enzymatic source is an important factor that can favor or hinder hydrolysis and transgalactosylation reactions. β-Galactosidases from *A. oryzae* and *Bacillus circulans* exhibit higher transgalactosylation activity than enzymes derived from *K. lactis* and *K. fragilis*, which tend to favor the hydrolysis reaction [20].

Guerreiro et al. [21] also investigated lactulose production using β-galactosidases from *(A) oryzae*, *K. lactis*, and *(B) circulans*, obtaining lactulose concentrations of approximately 20.0, 5.0, and 1.0 g·L⁻¹, respectively. The results reported by Guerreiro et al. [21] for β-galactosidase from *A. oryzae* (20.0 g·L⁻¹) are consistent with those obtained in the present study, which achieved a similar lactulose concentration (17.20 g·L⁻¹) using the same enzyme source.

GOS production yields were also determined. The highest yield was obtained with the *K. lactis* enzyme after six hours, reaching 17.58 ± 0.33%. For the enzyme, the maximum yield was 10.35 ± 0.57% after two hours of reaction. One of the main challenges in enzymatic lactulose synthesis is the typically low yield values reported in the literature [22]. Therefore, exploring strategies to enhance these yields is of considerable importance.

Fatahhi et al. [23] evaluated lactulose production using a commercial β-galactosidase from *K. lactis*. The reaction was carried out at pH 6.7 and 40 °C in a solution containing 120 g of lactose and 360 g of fructose. The maximum lactulose concentration reached 12.4 g·L⁻¹ after 120 min, corresponding to a yield of 10.5%. These findings highlight the potential of the adopted methodology, as in the present study, the maximum lactulose concentration obtained with β-galactosidase from *K. lactis* was 29.18 g·L⁻¹, with a yield of 17.58%.

We also evaluated lactulose synthesis using immobilized β-galactosidases from *K. lactis* and *A. oryzae*. The *K. lactis* enzyme was immobilized on controlled-porosity silica via covalent bonding, whereas the *A. oryzae* enzyme was immobilized by adsorption onto Duolite^®^ A-568 resin. Both enzymes exhibit distinct characteristics, such as optimal pH and temperature ranges; therefore, different immobilization methodologies were employed, as described.

As shown in Fig. 2C, K. *lactis* β-galactosidase immobilized on controlled-porosity silica did not produce lactulose during the six-hour reaction. After four hours, glucose and galactose began to appear as a result of lactose hydrolysis, and only 34.83% of the lactose was converted.

One possible explanation for the absence of lactulose formation is the small amount of immobilized support used (0.1 g per reaction). Therefore, further investigation focused on the *A. oryzae* β-galactosidase. Moreover, immobilization of *K. lactis* β-galactosidase requires a longer preparation time, and the use of silica as a support increases process cost compared to *A. oryzae* enzyme immobilization on Duolite^®^ A-568 resin. The resin also represents a more promising option for large-scale applications due to its favorable particle size and handling characteristics.

Using *A. oryzae* β-galactosidase (Fig. 2D), substrate consumption and product formation were observed after 0.5 h of reaction. Lactulose synthesis commenced after 2 h, reaching a maximum concentration of 23.46 ± 1.09 g·L⁻¹ at 4 h. A slight decline was observed thereafter (20.07 ± 0.96 g·L⁻¹), with the reaction ending at 11.62 ± 2.54 g·L⁻¹. The maximum yield was 14.13% after 2 h of reaction, and lactose conversion reached 93.24%.

A comparison of the two immobilized enzymes indicates that resin-immobilized *A. oryzae* β-galactosidase provides the best performance and shows the greatest potential for lactulose synthesis. Under the tested conditions, *K. lactis* β-galactosidase was unable to produce lactulose. Consequently, the subsequent investigations focused on optimizing the lactulose synthesis process using *A. oryzae* β-galactosidase.

**Fig. 3:**
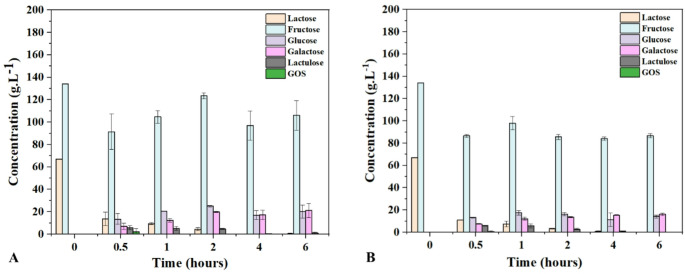
Concentration of products formed in the reaction with 200 g.L^− 1^ of initial sugars **A** free *A. oryzae* β-galactosidase, **B** *A. oryzae* β-galactosidase immobilized on Duolite ^®^ A-568 resin

Initial sugar concentrations of 200 were evaluated, and the results are presented in Fig. 3. Using the lower initial sugar concentration (200 g.L⁻¹) for both free and immobilized enzymes resulted in reduced lactulose production. With free *A. oryzae* β-galactosidase (Fig. 3A), substrate consumption was observed over time, with lactose reaching zero concentration after six hours. Lactulose formation began after 0.5 h, reaching a maximum concentration of 6.01 ± 0.29 g.L⁻¹ at that time. By the end of the reaction, the lactulose concentration had decreased to 1.09 ± 0.30 g.L⁻¹. Glucose and galactose, indicative of lactose hydrolysis, were also detected. Lactose conversion reached 100%.

At an initial sugar concentration of 500 g.L⁻¹, maximum lactulose synthesis reached 17.20 g.L⁻¹ after two hours of reaction (Fig. 2B). These results indicate that reducing the initial sugar concentration directly affects lactulose production, resulting in approximately a threefold decrease in lactulose concentration (from 6.01 g.L⁻¹ to 2.03 g.L⁻¹).

A similar reaction behavior was observed for the immobilized *A. oryzae* β-galactosidase. Almost complete lactose conversion (98.91%) was achieved within six hours of reaction. Glucose and galactose appeared after 0.5 h of reaction time, and during this period, 2.0 ± 2.8 g.L⁻¹ of GOS was also detected. Lactulose reached its maximum concentration (5.75 ± 1.72 g.L⁻¹) at 0.5 h, decreasing over time to 0.97 ± 0.99 g.L⁻¹ after six hours. The maximum lactulose concentration obtained at an initial sugar concentration of 200 g.L⁻¹ (5.79 g·L⁻¹) was approximately four times lower than that achieved at 500 g.L⁻¹ (23.46 g.L⁻¹).

Based on the above findings, it is evident that the initial sugar concentration in the reaction medium directly influences lactulose synthesis. Reducing the initial sugar concentration from 500 g.L⁻¹ to 200 g.L⁻¹ led to a three-to fourfold decrease in lactulose concentration. Faster lactulose formation was also observed at lower substrate concentrations; however, the highest initial sugar concentration yielded the greatest lactulose production, which occurred within approximately two hours of reaction.

Albuquerque et al. [19] evaluated lactulose production using β-galactosidase from *K. lactis*. The highest lactulose concentration (14.45 g.L⁻¹) was obtained with an initial sugar concentration of 200 g.L⁻¹. However, when the initial sugar concentration was increased to 450 g.L⁻¹, lactulose production decreased to 10.87 g.L⁻¹. These results contrast with those obtained in the present study using β-galactosidase from *A. oryzae*. The difference in enzyme source likely influenced the catalytic characteristics and overall performance of the process. One notable factor is the difference in substrate inhibition behavior between the two enzymes, which may have affected lactulose production at higher sugar concentrations in the study by Albuquerque et al. [19].

Lactulose yields were also calculated. The highest yield was obtained with the free *A. oryzae* β-galactosidase, reaching 9.00 ± 0.43%. For the immobilized enzyme, the maximum yield was 8.60 ± 2.6%. Both maximum yields were achieved within 0.5 h of reaction. After 6 h, the yields decreased, reaching 0% for the free enzyme and 1.44 ± 1.47% for the resin-immobilized enzyme.

Therefore, the optimal condition for lactulose synthesis was an initial sugar concentration of 500 g.L⁻¹. Reaction time was also an important factor. For *A. oryzae* β-galactosidase, two hours were sufficient to achieve maximum lactulose synthesis, after which lactulose concentrations decreased within six hours. This decrease can be attributed to lactulose consumption in subsequent reactions leading to the formation of other types of GOS. Consequently, the reaction time could be shortened. Shorter reaction times are advantageous in industrial applications, as they enable higher daily production rates and reduce equipment costs. Considering that *A. oryzae* β-galactosidase can tolerate higher temperatures, it would be interesting to investigate the process at an elevated temperature, such as 50 °C [18].

**Fig. 4:**
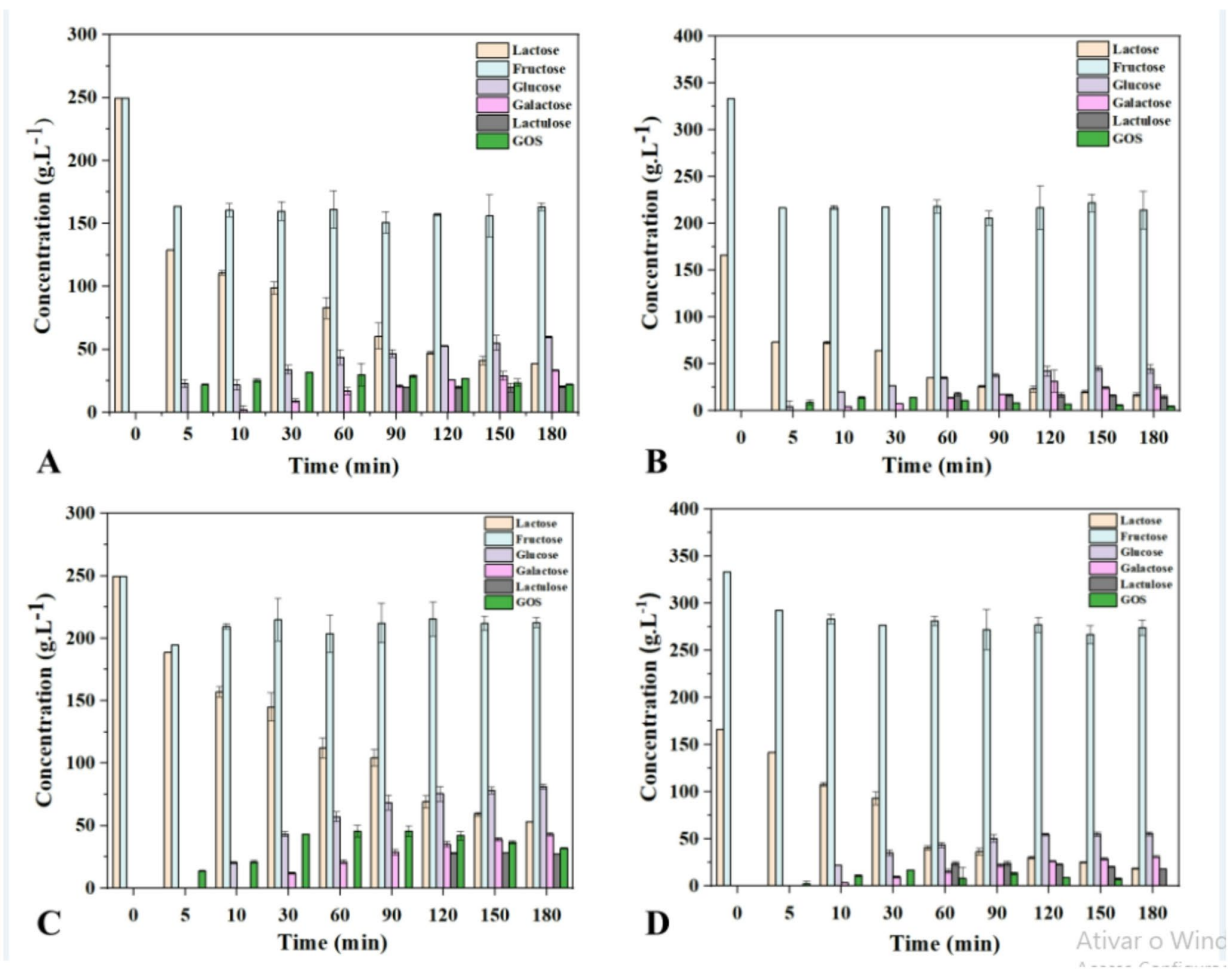
- The concentration of products formed in the reaction with 500 g.L^− 1^ of initial sugars and free and immobilized β-galactosidase from *A. oryzae* is shown below: **A** free enzyme with 1:1 ratio of lactose and fructose, **B** free enzyme with 1:2 ratio of lactose and fructose, **C** immobilized enzyme with 1:1 ratio of lactose and fructose, **D** immobilized enzyme with 1:2 ratio of lactose and fructose

The effect of the lactose-to-fructose ratio was also evaluated in the synthesis of lactulose using *A. oryzae* β-galactosidase in both its free and immobilized forms. Increasing the temperature from 40 to 50 °C enhanced lactulose synthesis and reduced the reaction time to three hours. Figure 4 shows the results obtained with the free *A. oryzae* β-galactosidase at lactose-to-fructose ratios of 1:1 and 1:2.

Lactulose was produced at both ratios when using the free enzyme. For the 1:1 lactose-to-fructose ratio (Fig. 4A), product formation was observed after five minutes of reaction. The detected products included glucose and galactose from lactose hydrolysis, as well as GOS. The total product concentration increased until 60 min, reaching a maximum of 31.92 ± 6.53 g.L⁻¹. After 180 min, the concentration decreased to 22.06 ± 0.63 g·L⁻¹. Lactulose appeared only after 90 min, reaching a concentration of 20.09 ± 1.11 g.L⁻¹, and remained stable until the end of the reaction. According to Tukey’s test, no significant differences were observed among the lactulose concentrations at 90, 120, 150, and 180 min (*p* < 0.05). The final lactose conversion was 84.55%.

A similar behavior was observed at the 1:2 lactose-to-fructose ratio (Fig. 4B), with product formation occurring after five minutes of reaction. GOS was detected throughout the process. Lactulose appeared after 60 min, reaching a maximum concentration of 17.91 ± 1.81 g.L⁻¹. This value was slightly lower (by 2.18 g.L⁻¹) than that obtained with the 1:1 lactose-to-fructose ratio. After 180 min, the lactulose concentration decreased to 14.75 ± 1.73 g.L⁻¹. The final lactose conversion was 89.84%.

Using the immobilized enzyme, lactulose synthesis was observed at both lactose-to-fructose ratios (Fig. 4C e D). For the 1:1 lactose-to-fructose ratio, significant GOS formation was detected from five minutes onward and continued throughout the reaction. The maximum GOS concentration was reached at 90 min, with a value of 45.72 ± 4.18 g.L⁻¹. Compared with previous results, this condition exhibited the highest GOS formation. Glucose and galactose were also detected as a result of lactose hydrolysis. Lactulose was first identified after 120 min, reaching a concentration of 28.15 ± 0.53 g.L⁻¹, which remained constant until 180 min. According to Tukey’s test at a 95% confidence level, no significant differences were observed among the lactulose concentrations at 120, 150, and 180 min. Compared with previous results, lactulose synthesis occurred more slowly with the immobilized enzyme. The final lactose conversion was 78.12%.

Lower GOS production was observed at the 1:2 lactose-to-fructose ratio (Fig. 4D) compared with the 1:1 ratio. Under these conditions, lactulose synthesis occurred more rapidly, being detected within 60 min of reaction. The maximum lactulose concentration reached 24.04 ± 2.17 g.L⁻¹ at 90 min. Glucose and galactose concentrations increased steadily from 5 to 180 min. At the end of the process, lactose conversion reached 88.91%.

Lactulose yield (Y_Lu_) was calculated for all conditions. Table 1 presents the lactulose concentrations and yields corresponding to each evaluated condition.

**Table 1:** Lactulose concentration and yield for free and immobilized *A. Oryzae* β-Galactosidase in media with lactose-to-fructose ratios of 1:1 and 1:2

	Time (min)	Ratio (1:1) of lactose and fructose		Ratio (1:2) of lactose and fructose	
		Lactulose Concentration (g.L^− 1^)	Lactulose yield (%)	Lactulose Concentration (g.L^− 1^)	Lactulose yield (%)
Free β-gal from *A. oryzae*	60	0.00	0.00	17.91 ± 1.81^c^	10.79 ± 1.09^d^
	90	20.09 ± 0.00^a^	8.05 ± 0.00^b^	16.44 ± 1.00^c^	9.91 ± 0.61^d^
	120	20.09 ± 1.11^a^	8.05 ± 0.45^b^	16.77 ± 2.10^c^	10.11 ± 1.26^d^
	150	19.77 ± 3.30^a^	7.92 ± 1.32^b^	16.35 ± 0.93^c^	9.84 ± 0.56^d^
	180	20.47 ± 0.84^a^	8.21 ± 0.34^b^	14.75 ± 1.73^c^	8.89 ± 1.04^d^
Immobilized *A. oryzae* from β-gal	60	0.00	0.00	23.85 ± 2.04^g^	14.37 ± 1.23^i^
	90	0.00	0.00	24.04 ± 2.17^g^	14.48 ± 1.31^i^
	120	28.15 ± 0.53^e^	11.28 ± 0.21^f^	22.86 ± 0.76^gh^	13.77 ± 0.46^ij^
	150	28.36 ± 0.27^e^	11.37 ± 0.11^f^	20.52 ± 0.63^gh^	12.36 ± 0.38^ij^
	180	27.56 ± 0.06^e^	11.04 ± 0.02^f^	18.01 ± 0.55^g^	10.85 ± 0.33^i^

Table 1 presents the consolidated results of lactulose production. The highest lactulose concentration was generally achieved with *A. oryzae* β-galactosidase immobilized at a 1:1 lactose-to-fructose ratio (28.15 g.L⁻¹), whereas the highest yield was obtained with the enzyme immobilized at a 1:2 lactose-to-fructose ratio (14.48%). Considering the inherent challenges of enzymatic synthesis and literature reports, all lactulose productions were considered reasonable despite the observed differences. Guerrero et al. [21] reported the maximum lactulose concentration at a lactose-to-fructose ratio of 1:2, reaching 15.25 ± 0.52 g.L⁻¹. Compared with the results obtained in the present study, both the free and immobilized enzyme produced higher lactulose concentrations at the 1:1 and 1:2 ratios than the value reported by Guerrero et al. [21].

Although the highest lactulose concentration was achieved at a 1:1 lactose-to-fructose ratio, the standard condition for the methodology was established at 1:2. This ratio resulted in higher lactulose yields while taking by-product formation into account. Using a 1:1 ratio favored GOS production over lactulose synthesis, a behavior also reported by Guerrero et al. [21]. Albuquerque et al. [19] investigated lactulose synthesis using β-galactosidase from *K. lactis* and similarly determined that the optimal lactose-to-fructose ratio is 1:2, using a mixture of 15% (m/v) lactose and 30% (m/v) fructose, which yielded a lactulose concentration of 10.34 g.L⁻¹.

Table 2 compares the results obtained in the present study with those reported in other studies that aimed to enzymatically synthesize lactulose using β-galactosidase.

**Table 2:** Lactulose synthesis under different conditions using various β-galactosidase sources

Enzyme Source	Substrate (g.L^− 1^)		Free or immobilized enzyme	Lactulose concentration (g.L^− 1^)	Lactulose yield (%)	References
	Lactose	Fructose				
*A. oryzae*	166.0	333.0	Immobilized	24.04	14.48	Presente Estudo
*K. lactis*	66.7	133.3	Free	14.50	20.00	[19]
*K. lactis*	136.8 a 182.4	422.5 a 507.0	Free	8.36	6.00	[24]
*L. plantarum FMNP01*	400	200	Free	18.32	--	[25]
*K. lactis*	66.6	133.2	Immobilized	11.00	--	[13]

The potential of *A. oryzae* β-galactosidase as a lactulose producer is promising. Nevertheless, there remains room for improvement to achieve higher lactulose concentrations and yields. Therefore, continued research and further studies are essential to advance the enzymatic lactulose synthesis pathway and facilitate its adoption as a profitable industrial process.

### Synthesis of lactulose in a jacketed, stirred tank reactor with magnetic stirring

Using immobilized *K. lactis* β-galactosidase for lactulose production proved challenging in an orbital-stirred reactor; nevertheless, this reactor configuration was evaluated. With free *K. lactis* β-galactosidase, product formation including glucose and galactose from lactose hydrolysis as well as lactulose synthesis did not begin until 60 min of reaction. The highest lactulose concentration, 37.20 ± 0.03 g.L⁻¹, was reached after 180 min. When compared with the concentrations obtained in a conical reactor with orbital stirring (29.18 ± 0.55 g.L⁻¹), lactulose production was higher in the stirred-tank reactor with magnetic stirring. Lactose conversion at the end of the process was 73.44%.

Upon analyzing the reaction with immobilized *K. lactis* β-galactosidase, no product formation was observed; neither lactose hydrolysis nor transgalactosylation for lactulose synthesis occurred. The concentrations of the substrates, lactose and fructose, varied slightly throughout the reaction, likely due to experimental errors.

Lactulose synthesis was also not achieved using a conical reactor with orbital stirring for the immobilized enzyme. However, glucose and galactose were detected at the end of the reaction, resulting from lactose hydrolysis. These results indicate that lactulose synthesis using immobilized *K. lactis* β-galactosidase on controlled-porosity silica is challenging, whereas the free enzyme demonstrated effective lactulose production.

**Fig. 5:**
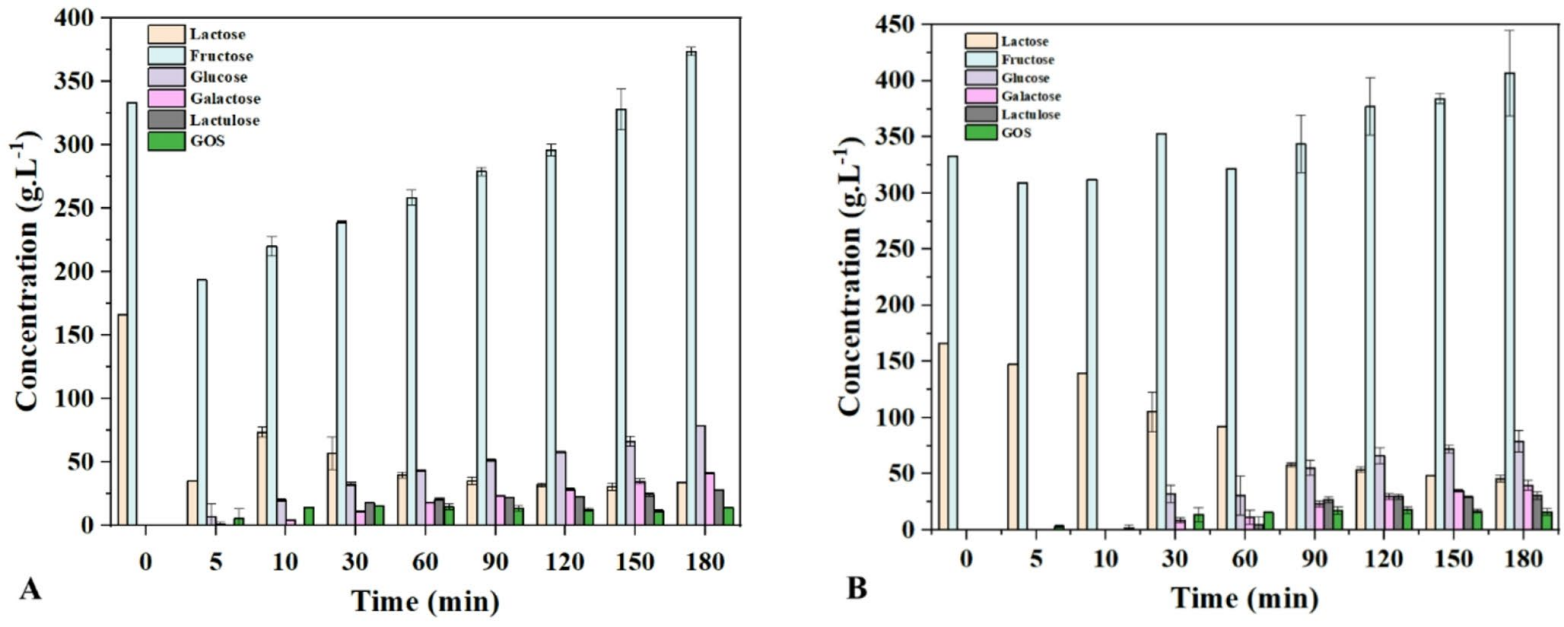
Product concentration in a reaction with a jacketed reactor with magnetic stirring: **A** Free *A. oryzae* β-galactosidase, **B** Immobilized *A. oryzae* β-galactosidase

Lactulose synthesis was additionally evaluated using *A. oryzae* β-galactosidase in a jacketed, stirred-tank reactor with magnetic stirring. The results are presented in Fig. 5.

The results obtained with free and immobilized *A. oryzae* β-galactosidase exhibited different substrate behavior compared with previous results using a conical reactor with orbital stirring. As expected, lactose levels decreased due to consumption. Interestingly, fructose concentrations initially decreased during the first five minutes of the reaction, then increased throughout the process, surpassing the initial levels after 180 min. This behavior may be attributed to the effects of temperature and magnetic stirring, with the open jacketed reactor potentially contributing to the observed changes. Despite this unexpected trend, product formation was observed.

Figure 5A illustrates the formation of glucose, galactose, and GOS after five minutes for free *A. oryzae* β-galactosidase. Lactulose synthesis commenced after 30 min and continued to increase until 180 min, reaching a maximum concentration of 27.78 ± 0.21 g.L⁻¹. When compared with the lactulose concentration obtained using a conical reactor with orbital stirring (20.09 g.L⁻¹), it is evident that the reactor with magnetic stirring yielded a higher lactulose concentration. Lactulose conversion reached 79.58%.

Similar behavior was observed for the immobilized β-galactosidase from *A. oryzae* in Fig. 5B, where product formation began after five minutes of reaction and increased until the end of the process. Glucose, galactose, GOS, and lactulose were formed. Lactulose synthesis commenced after 90 min, reaching a maximum concentration of 30.85 ± 3.26 g.L⁻¹ at the end of the reaction. This concentration can be compared with that obtained using a conical reactor with orbital stirring (24.04 g.L⁻¹), representing an increase of 6.81 g.L⁻¹ when using the reactor with magnetic stirring. Lactulose conversion reached 79.58%.

Shen et al. [26] evaluated lactulose synthesis using β-galactosidase from *K. lactis* under orbital shaking conditions and reported a maximum lactulose concentration of 15.40 g.L⁻¹. The results obtained in the present study were higher, reaching approximately 29.27 g.L⁻¹ across the three evaluated conditions.

Lactulose yields were calculated for free *K. lactis* β-galactosidase as well as for free and immobilized *A. oryzae* β-galactosidase. Notably, no lactulose synthesis was detected for silica-immobilized *K. lactis* β-galactosidase. For all three enzymes, yields increased over time, peaking at the end of the reaction. The highest lactulose yield observed in this reactor configuration was 22.41 ± 0.002% for free *K. lactis* β-galactosidase. Among the free and immobilized *A. oryzae* β-galactosidase, the immobilized enzyme exhibited the highest yield (18.58 ± 1.96%).

Overall, the jacketed reactor with magnetic stirring proved to be an efficient setup for lactulose synthesis, generally producing higher concentrations than the conical reactor with orbital stirring.

### Synthesis of lactulose in a fixed-bed reactor

**Fig. 6:**
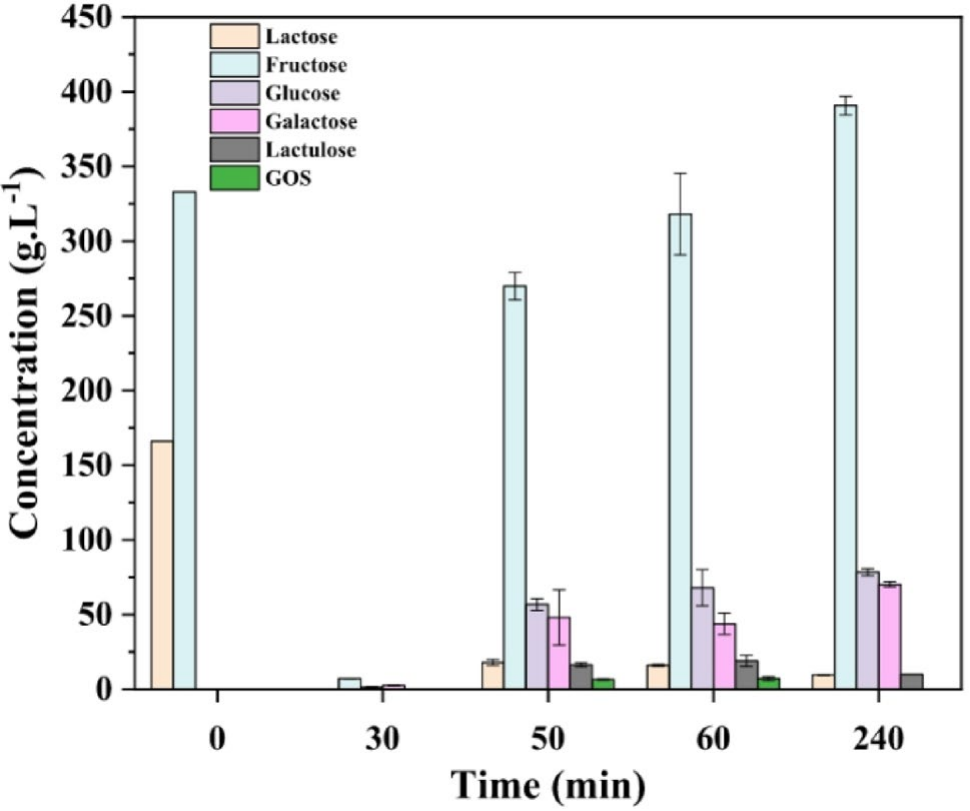
Concentrations of Products Obtained during Lactulose Synthesis in a Fixed-Bed Reactor

To enhance lactulose synthesis and enable process scale-up, a fixed-bed reactor was evaluated using immobilized *A. oryzae* β-galactosidase. The reactor was packed with 5.0 g of immobilized biocatalyst and 30.0 g of glass beads as an inert support material. The flow rate was maintained at 1 mL.min⁻¹. The process was conducted in two stages: an initial continuous mode, followed by a fed-batch operation with recycling of the reaction mixture for three hours. The results are presented in Fig. 6.

Figure 6 illustrates the two stages of the fixed-bed process. The times up to 60 min correspond to the continuous operation regime, while the 240 min mark represents the results obtained under product recycling conditions. Regarding the substrates lactose and fructose, a low fructose concentration was observed at 30 min of reaction time, followed by an increase until the end of the reaction. A similar trend was observed in previous results for the stirred-tank reactor with magnetic stirring (Fig. 5). After 240 min, the fructose concentration reached 390.68 ± 6.18 g.L⁻¹, which is higher than the initial concentration of 333.33 g.L⁻¹.

A high rate of lactose hydrolysis was observed, producing 78.40 ± 2.44 g.L⁻¹ of glucose and 70.13 ± 1.71 g.L⁻¹ of galactose by the end of the process. Lactulose synthesis commenced after 30 min and reached its maximum after 60 min. The continuous process concluded with 19.02 ± 3.79 g.L⁻¹ of lactulose and a yield of 11.46 ± 2.28%. In contrast, the process with product recycling resulted in a lower final lactulose concentration of 9.93 ± 0.04 g·L⁻¹ and a yield of 5.98 ± 0.02%. This maximum lactulose concentration (19.02 g·L⁻¹) was lower than that obtained with previous reactor configurations, which reached approximately 30.0 g·L⁻¹. Although the fixed-bed reactor was expected to enhance lactulose synthesis, further optimization of the process conditions is required, including feed flow rate, the amount of immobilized biocatalyst and inert material, reaction time, and other relevant variables.

Guerreiro et al. [27] evaluated a continuous process for lactulose production using *A. oryzae* β-galactosidase immobilized on glyoxyl agarose. The best results were achieved with 50% (m/m) total sugars, a fructose/lactose molar ratio of 12, a flow rate of 15.0 mL.min⁻¹, and a biocatalyst-to-inert mass ratio of 1:8. They reported a maximum yield of 0.6 g of lactulose per g of initial lactose and a lactose conversion of 28%. However, compared with previous results, the lactulose concentration per initial lactose was lower, at 0.11 g of lactulose per g of initial lactose.

In summary, the fixed-bed reactor process shows promise; however, further investigation is required to improve lactulose concentrations and yields.

## Conclusions

This study demonstrated the feasibility of various reactor configurations for lactulose synthesis. Both free *K. lactis* β-galactosidase and free and immobilized *A. oryzae* β-galactosidase were effective in producing lactulose. The optimal conditions for synthesis were identified as an initial sugar concentration of 500.0 g·L⁻¹, a lactose-to-fructose molar ratio of 1:2, and temperatures of 50 °C for *A. oryzae* β-galactosidase and 40 °C for *K. lactis* β-galactosidase. Among the reactor systems evaluated, the conical reactor with orbital stirring yielded lactulose concentrations of 20.0–28.0 g·L⁻¹. The stirred-tank reactor with magnetic stirring achieved slightly higher concentrations, approximately 30.0 g·L⁻¹. The fixed-bed reactor produced a maximum lactulose concentration of 19.02 g·L⁻¹, highlighting the need for further optimization of this configuration. Overall, the results emphasize the potential of enzymatic lactulose synthesis and provide a foundation for future process integration and scale-up studies.

## Data Availability

Data will be made available on request.

## References

[1] Wang M, Wang L, Lyu X, Hua X, Goddard JM, Yang R (2022) Lactulose production from lactose isomerization by chemo-catalysts and enzymes: Current status and future perspectives. Biotechnol Adv v 60. 10.1016/j.biotechadv.2022.108021 10.1016/j.biotechadv.2022.108021 35901861

[2] Yang X, Zeng D, Li C, Yu M, Xie G, Zhang Y, Lu W (2023) Therapeutic potential and mechanism of functional oligosaccharides in inflammatory bowel disease: a review. Food Sci Hum Wellness v 12(6):2135–2150. 10.1016/j.fshw.2023.03.027

[3] Montilla A, Castillo MD, Sanz ML, Olano A (2005) Egg shell as catalyst of lactose isomerization to lactulose. Food Chemistry, v. 90, pp. 883–890, 10.1016/j.foodchem.2004.05.042

[4] Nagasawa T, Katsuyuki S, Kasumi T (2019) Efficient Continuous Production of Lactulose Syrup by Alkaline Isomerization Using an Organogermanium Compound. Journal of Applied Glycoscience, v. 66, pp. 121–129. 10.5458/jag.jag.JAG-2019_0012 10.5458/jag.jag.JAG-2019_0012 PMC8373580 34429690

[5] Weber LZ, Moreira CLO, Krieger N, Mitchel DA (2024) Model-based estimation of selectivities of the β-galactosidase of *Aspergillus oryzae* in the production of lactulose and fructosyl-galactooligosaccharides. Biochem Eng J v 209. 10.1016/j.bej.2024.109408

[6] Huerta M, Martín AS, Arancibia B, Cornejo FA, ARENAS F, Illanes A, Guerrero C, Vera C (2024) Integrating the enzymatic synthezes of lactulose, epilactose and galacto-oligosaccharides. Food and Bioproducts Processing, v. 147, pp. 474–482, 10.1016/j.fbp.2024.08.002

[7] Panagopoulos V, Karabagias IK, Dima A, Boura K, Kanellaki M, Bosnea L, Nigam PSNN, Koutinas A (2024) Promotion of lactose isomerization to fructose and lactulose in one batch by immobilized enzymes on bacterial cellulose membranes. Food Chem v 457. 10.1016/j.foodchem.2024.140127 10.1016/j.foodchem.2024.140127 38908252

[8] Lu L, Guo L, Wang K, Liu Y, Xiao M (2020) β-Galactosidases: A great tool for synthesizing galactose-containing carbohydrates. Biotechnol Adv v 39. 10.1016/j.biotechadv.2019.107465 10.1016/j.biotechadv.2019.107465 31689470

[9] Sabater C, Fara A, Palacios J, Corzo N, Requena T, Montilla A, Zárate G (2019) Synthesis of prebiotic galactooligosaccharides from lactose and lactulose by dairy propionibacteria. Food Microbiol 77:93–105. 10.1016/j.fm.2018.08.014 30297061 10.1016/j.fm.2018.08.014

[10] Yin H, Bultema JB, Dijkhuizen L, Leeuwen SSV (2017) Reaction kinetics and galactooligosaccharide product profiles of the β-galactosidases from *Bacillus circulans, Kluyveromyces lactis* and *Aspergillus oryzae*. Food Chem 225:230–238. 10.1016/j.foodchem.2017.01.030 28193420 10.1016/j.foodchem.2017.01.030

[11] Vera C, Guerrero C, Aburto C, Cordova A, Illanes A (2020) Conventional and non-conventional applications of β-galactosidases. Biochim Biophys Acta (BBA) - Proteins and Proteomics, v. 1868, pp. 1–12, 10.1016/j.bbapap.2019.140271 10.1016/j.bbapap.2019.140271 31494342

[12] Sirisha VL, Jain A, Jain A (2016) Enzyme immobilization: An overview on methods, support material, and applications of immobilized enzymes. Adv Food Nutr Res 1–33. 10.1016/bs.afnr.2016.07.004 10.1016/bs.afnr.2016.07.004 27770861

[13] Girão Neto CAC, Silva NCG, Costa TO, Albuquerque TL, Gonçalves LRB, Fernandez-Lafuente R, Rocha MV (2021) P. The β-galactosidase immobilization protocol determines its performance as catalysts in the kinetically controlled synthesis of lactulose. Int J Biol Macromol v 176:468–478. 10.1016/j.ijbiomac.2021.02.078 10.1016/j.ijbiomac.2021.02.078 33592268

[14] Ramiréz N, Ubilla C, Campos J, Valencia F, Aburto C, Vera C, ILLANES A, GUERRERO C (2021) Enzymatic production of lactulose by fed-batch and repeated fed-batch reactor. Bioresource Technol v 341. 10.1016/j.biortech.2021.125769 10.1016/j.biortech.2021.125769 34416660

[15] Serey M, Vera C, Guerrero C, Illanes A (2021) Immobilization of Aspergillus oryzae β-galactosidase in cation functionalized agarose matrix and its application in the synthesis of lactulose. Int J Biol Macromol v 167:1564–1574. 10.1016/j.ijbiomac.2020.11.110 10.1016/j.ijbiomac.2020.11.110 33217465

[16] Sousa CC, Falleiros LNSS, RIbeiro EJ, Resende MM (2025) Immobilization of β-galactosidase of *Kluyveromyces lactis* in mesoporous sílica. Food Bioprod Process v 149:165–175. 10.1016/j.fbp.2024.11.011

[17] Bao J, Furumoto K, Fukunaga K, Nakao KA, Koumatsu K, Yoshimoto M (2004) Deactivation kinetics of immobilized glucose oxidase for production of calcium gluconate in an external loop airlift bioreactor. Biochem Eng J v 22:33–41. 10.1016/j.bej.2004.08.001

[18] Falleiros LNSS, Cabral BV, Fischer J, Guidini CZ, Cardoso VL, Resende MM, Ribeiro EJ (2017) Improvement of recovered activity and stability of the *Aspergillus oryzae* β-galactosidase immobilized on duolite^®^ A568 by combination of immobilization methods. Chem Industry&Chemical Eng Q 1–10. 10.2298/CICEQ160912010F

[19] Albuquerque TL, Gomes SDL, D’Almeida AP, Fernandez-Lafuente R, Gonçalves LRB, Rocha MVP (2018) Immobilization of β-galactosidase in glutaraldehyde-chitosan and its application to the synthesis of lactulose using cheese whey as feedstock. Process Biochem v 73:65–73. 10.1016/j.procbio.2018.08.010

[20] Albuquerque TL, Sousa M, Silva NCG, Girão Neto CAC, Gonçalves LRB, Fernandez-Lafuente R, Rocha M (2021) V. P. β-Galactosidase from *Kluyveromyces lactis*: Characterization, production, immobilization and applications - A review. Int J Biol Macromol v 191:881–898 10.1016/j.ijbiomac.2021.09.133 34571129

[21] Guerrero C, Vera C, Plou F, Illanes A (2011) Influence of reaction conditions on the selectivity of the synthesis of lactulose with microbial β- galactosidases. J Mol Catal B: Enzymatic v 72:206–212. 10.1016/j.molcatb.2011.06.007

[22] Vera C, Illanes A, Guerreiro C (2021) Enzymatic production of prebiotic oligosaccharides. Curr Opin Food Sci v 37:160–170. 10.1016/j.cofs.2020.10.013

[23] Fattahi H, Ashtiani FZ, Bonakdarpour B, Hashemi SA, KhatamI SH (2010) Enzymatic synthesis of lactulose by commercial β-galactosidase from *Klyveromyces lactis*. Afinidad v 67(546):149–153

[24] Khatami S, Ashtiani FZ, Bonakdarpour B, Mehrdad M (2014) The enzymatic production of lactulose via transglycosylation in conventional and non-conventional media. Int Dairy J 34:74–79. 10.1016/j.idairyj.2013.07.010

[25] Liao X, Zheng Q, Zhoud Q, Lin J, Guo L, Yun F (2016) Characterization of recombinant β-galactosidase and its use in enzymatic synthesis of lactulose from lactose and fructose. J Mol Catal B: Enzymatic v 134:253–260

[26] Shen Q, Yang R, Hua X, Ye F, Wang H, ZhaO W, Wang K (2012) Enzymatic synthesis and identification of oligosaccharides obtained by transgalactosylation of lactose in the presence of fructose using β-galactosidase from *Kluyveromyces lactis*. Food Chem 135:1547–1554. 10.1016/j.foodchem.2012.05.115 22953892 10.1016/j.foodchem.2012.05.115

[27] Guerrero C, Valdivia F, Ubilla C, Ramírez N, Gómez M, Aburto C, Vera C, Illanes A (2019) Continuous enzymatic synthesis of lactulose in packed-bed reactor with immobilized *Aspergillus oryzae* β-galactosidase. Bioresource Technol v 278:296–302. 10.1016/j.biortech.2018.12.018 10.1016/j.biortech.2018.12.018 30708333

